# Supplementary material for: Spiral ganglion neuron degeneration in aminoglycoside-deafened rats involves innate and adaptive immune responses not requiring complement
Source: Front Mol Neurosci. 2024 May 22;17:1389816. doi: 10.3389/fnmol.2024.1389816 (PMC11151750; doi:10.3389/fnmol.2024.1389816)
Supplement: FIGURE S1 — Experimental procedures. (A) Schematic depicting the dissection procedure for collecting tissue for RNA. The cochlea was isolated from the temporal bone and the bony capsule removed. The organ of Corti was then removed, leaving the spiral ganglion and associated cells. Cochleae were collected and RNA extraction performed as described in Materials and Methods. (B) Representative midmodiolar section of the cochlea. The different half turns are outline and labeled in green. (C) Western blot of liver protein lysates to detecting C3 protein in WT and heterozygous rats and confirming lack of C3 protein in C3KO rats. KM, kanamycin; ABR, auditory brainstem response; SGN, spiral ganglion neuron. [file Data_Sheet_1.PDF]

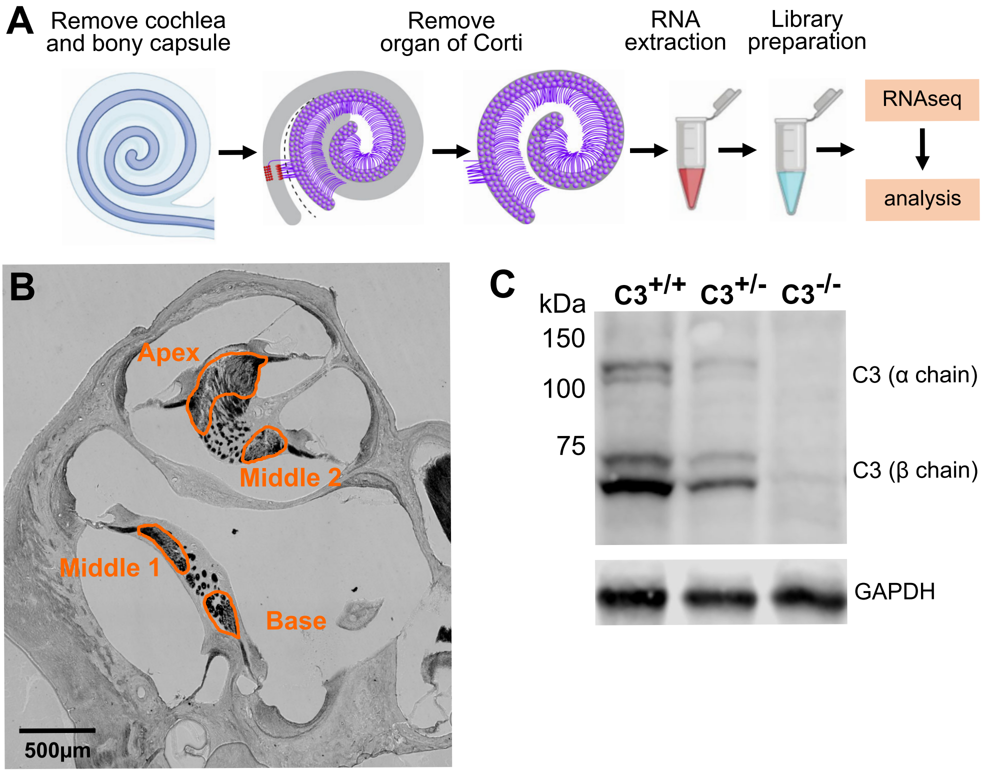

Supplementary Figure 1

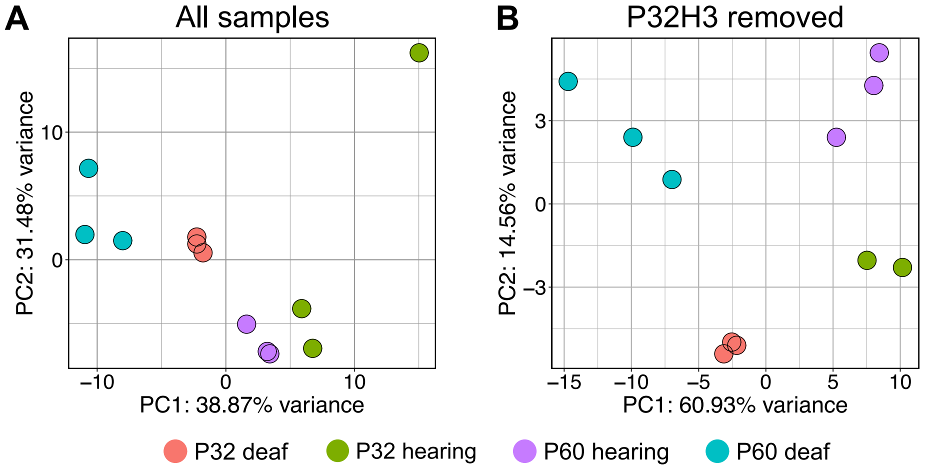

Supplementary Figure 2

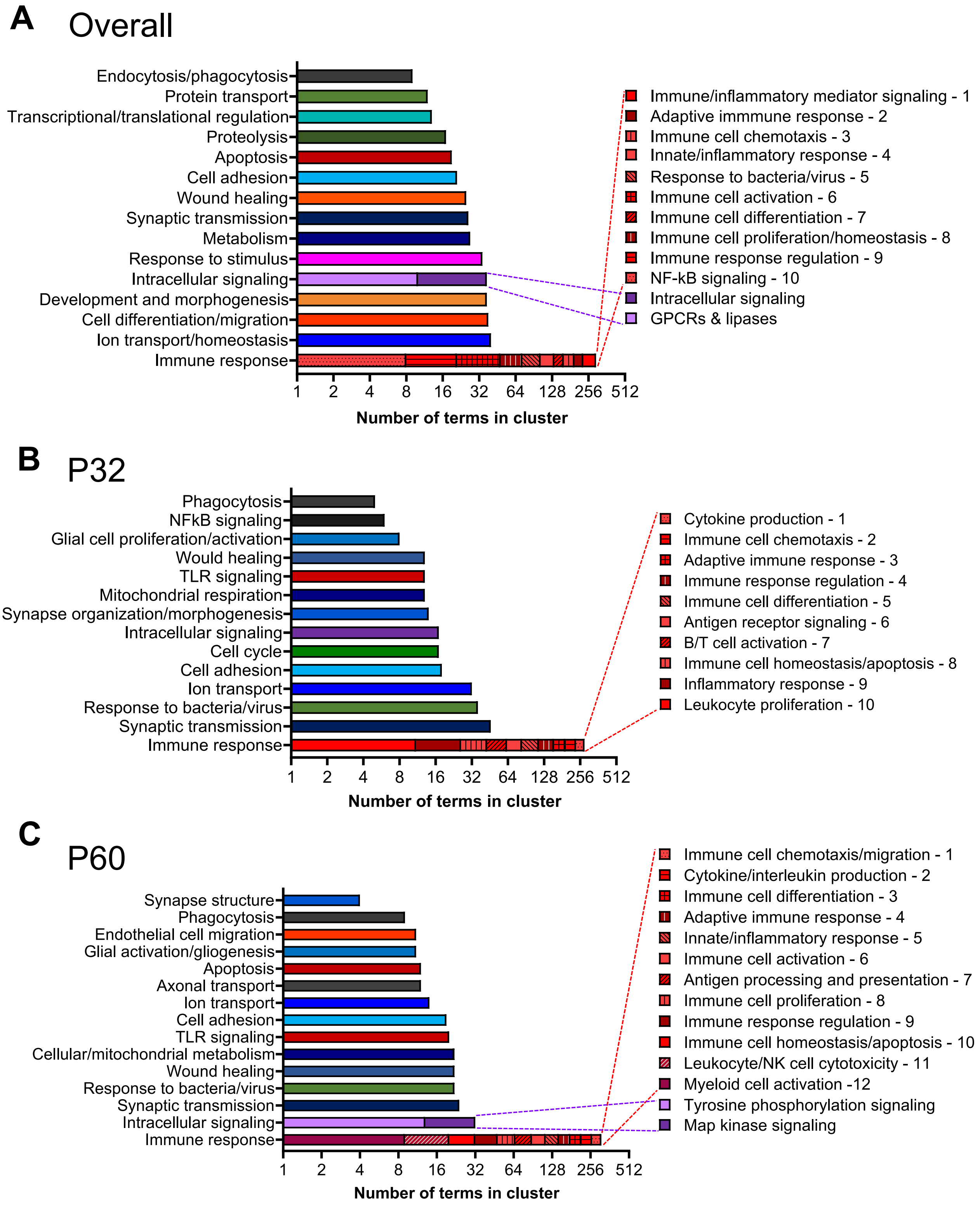

Supplementary Figure 3

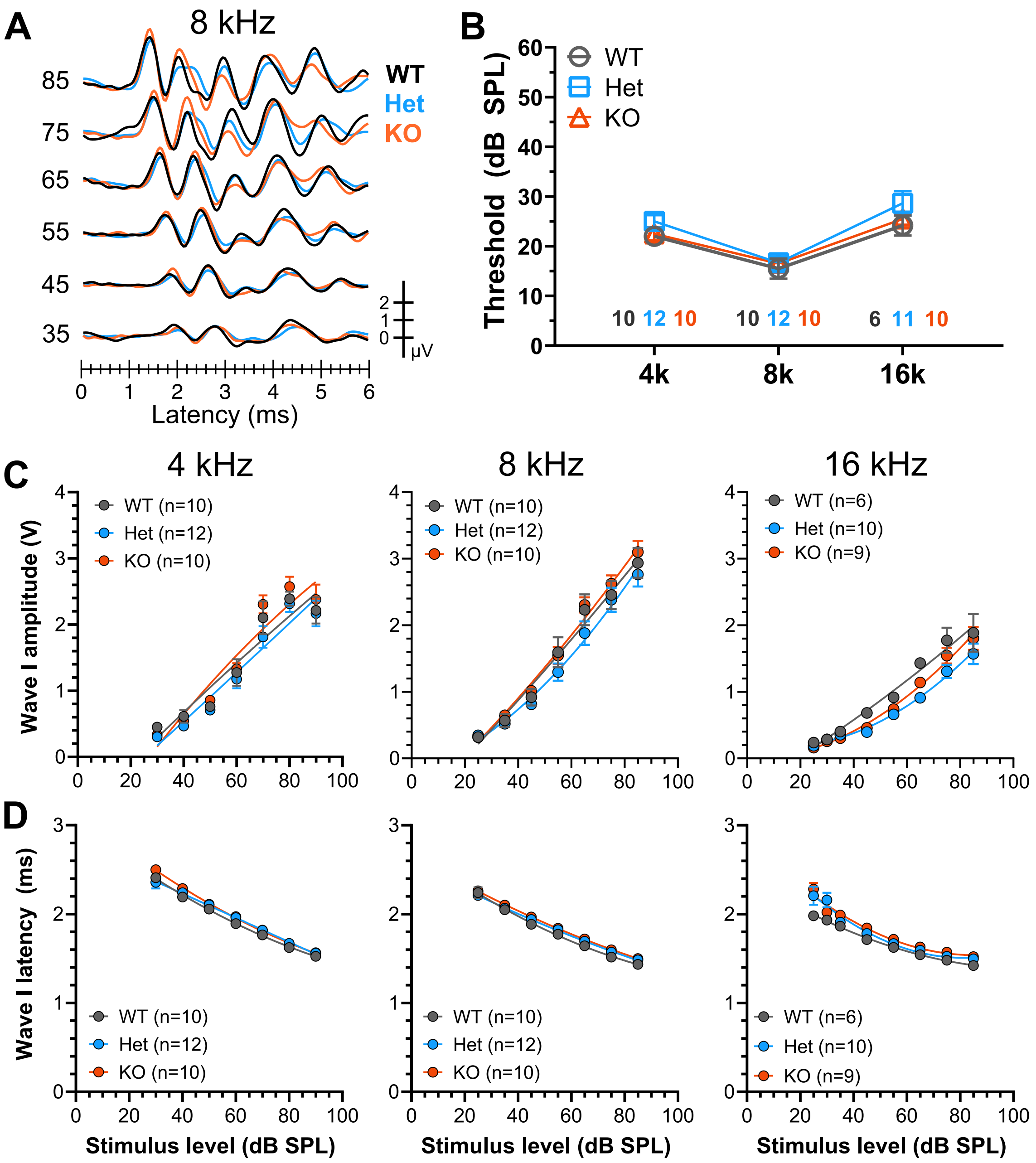

Supplementary Figure 4

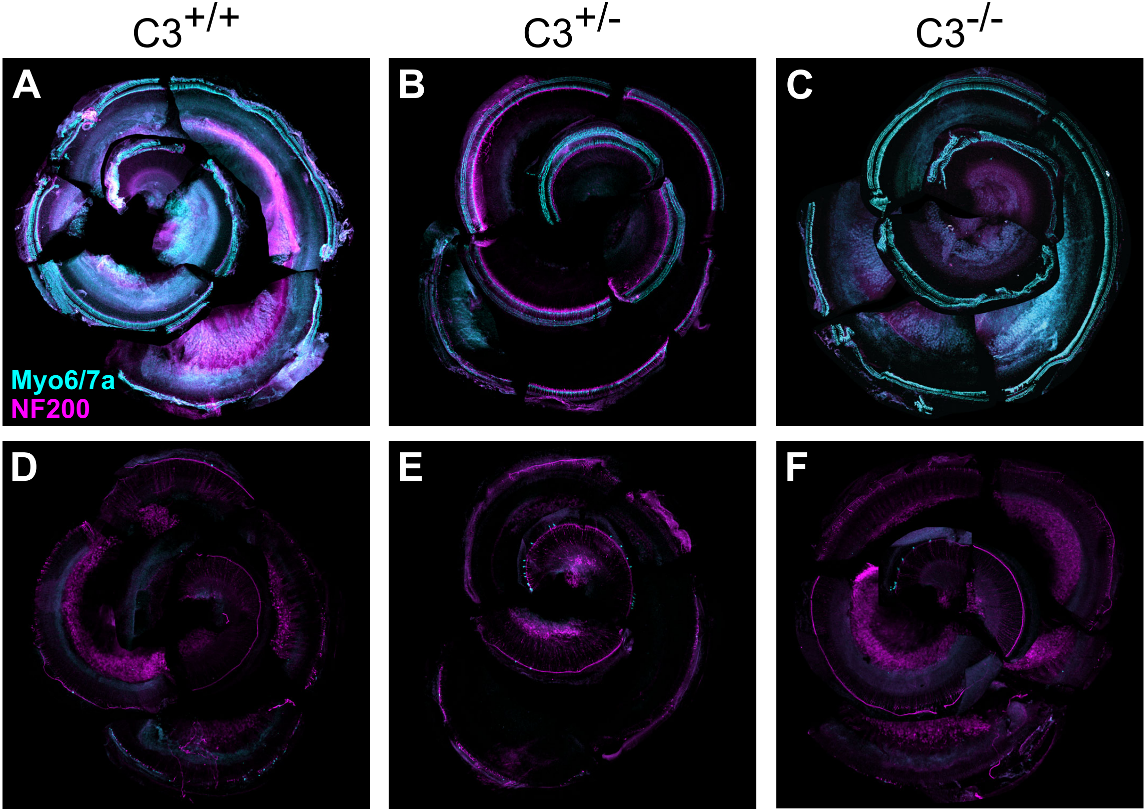

Supplementary Figure 5. Cochlear wholemounts labeled to show hair cells and neurons. Hair cells are labeled with Myo6/7a, neurons with NF200 antibodies. Genotypes are shown above each column: **A&D**, wildtype; **B&E**, heterozygotes; **C&F**, homozygous C3 knockouts. **A-C**, control undeafened; **D-F**, deafened with kanamycin.

Supplementary Figure 5

Supplementary Tables

Supplemental Table 1. qPCR primers

Gene name	Primer sequences	Accession ID	Amplicon length (bp)
C1qa	F: CCGGGTCTCAAAGGAGAGAG<br>R: CCAGATTCCCCCATGTCTC	NM_001008515.1	89
C3	F: CAGTGTGGGTGGATGTGAAG<br>R: GGCTGTCGGTTATCTCTTGG	NM_016994.2	76
Cd4	F: CCCTGAACCAGAAGAAGCAC<br>R: CAAGGTTGAGTGGGAAGGAG	NM_012705.1	123
Cd68	F: CTCATTCCCTTACGGACAGC<br>R: GCTGAGAATGTCCACTGTGC	NM_001031638.1	135
Cxcl10	F: GCTTATTGAAAGCGGTGAGC<br>R: GGGTAAAGGGAGGTGGAGAG	NM_139089.1	122
RT1-Da	F: AAGAAAATGGCCACACTTGG<br>R: CGGGGGAAAGATAGAACTCC	NM_001008847.2	130
Kcnc1	F: CCTACTCATCCCGCTATGC<br>R: GTGGGTTCTCAGACAGAAGG	NM_012856.2	89
Kcnc3	F: ACTGGGAATTATGGGATTGC<br>R: AGTCCTCATGAGCCAGTGC	NM_053997.5	140
Kcnd2	F: AGCCTTCTGGTACACCATCG<br>R: TTCCCTGCTATGGTTTTTGG	NM_031730.2	75
Nefh	F: CCGTCATCAGGTAGACATGG<br>R: AGCAGGTCCTGGTACTCTCG	NM_012607.2	117
Scn4b	F: GAACCGAGGCAAATACTCAGG<br>R: CAGATACCTCCAACGACAGG	NM_001008880.2	89
Scn8a	F: AGCGCTGAGAACATTCAGG<br>R: CTTCACGGACTGGATTAGGG	NM_019266.3	97
Syp	F: CCAGACAGGGAACACATGC<br>R: AGCCTGTCTCCTTGAACACG	NM_012664.3	131
Rps16	F: GAAGGGTGGTGGTCATGTG<br>R: TCCGATCGTACTGGATGAGG	NM_001169146.1	137

Supplemental Table 2. All of the primary antibodies used in this study are listed with the corresponding secondary antibodies and fluorophores typically used for visualization. All secondary antibodies are Invitrogen Alexa Fluor used at a dilution of 1:400.

cell type	antigen	primary antibody	dilution	vendor	cat. #	secondary Ab	fluorophore	cat. #
neurons	β_{III}-tubulin	TuJ1, mouse IgG2a	1:400	Biolegend	80122	anti mouse IgG2a	Alexa Fluor 633	A21136
macrophages	Iba1	rabbit polyclonal	1:800	Wako	019-19741	anti rabbit IgG	Alexa Fluor 488	A11008
activated macrophages	CD68	ED1, mouse IgG1	1:400	Abcam	31360	anti mouse IgG1	Alexa Fluor 546	A21123
hair cells	myosin VI and myosin VIIa	rabbit polyclonal	1:400<br>1:400	Sigma<br>Proteus	KA-15<br>25-6790	anti rabbit IgG	Alexa Fluor 488	A11008
immune cells	rat CD45	OX-1, mouse IgG1	1:100	Biolegend	202201	anti mouse IgG1	Alexa Fluor 546	A21123
MHCII-expressing cells	MHCII	OX-6, mouse IgG1	1:100	Biolegend	50-165-477	anti mouse IgG1	Alexa Fluor 546	A21123
